# Cost comparison of community-based vector surveillance using VectorCam^TM^, an AI-enabled mosquito identification tool, versus routine entomological surveillance in Uganda

**DOI:** 10.1186/s12936-026-06029-1

**Published:** 2026-08-01

**Authors:** Marina Rincon Torroella, Shreya Raman, Bryan N. Patenaude, Sunny Patel, Aryaman Shodhan, Atul Zacharias, Winnifred Kansiime, Catherine Maiteki, Jimmy Opigo, Peter Waiswa, Soumyadipta Acharya

**Affiliations:** 1 Center for Bioengineering Innovation and Design, Department of Biomedical Engineering, Whiting School of Engineering, The Johns Hopkins University, Baltimore, MD 21218 USA; 2 Bloomberg School of Public Health, The Johns Hopkins University, Baltimore, MD 21218 USA; 3 Ugandan Ministry of Health, National Malaria Elimination Division, Kampala, Uganda; 4 Makerere School of Public Health, Makerere University, Kampala, Uganda

**Keywords:** Artificial intelligence, Cost analysis, Digital health, Vector surveillance, Malaria control, Task-shifting, Convolutional neural networks, Community health workers, VectorCam^TM^, Health systems strengthening

## Abstract

**Background:**

National malaria elimination programs depend on timely and reliable vector surveillance data to guide interventions. Current systems rely on analog data collection and microscopy-based morphological identification of mosquitoes by entomologists (vector control officers, VCO), a process constrained by limited entomologists, high specimen volumes, and specimen transport from rural collection sites. VectorCam^TM^ is a smartphone-based device that uses AI to classify mosquito species, sex, and abdominal status within seconds, enabling automated workflows and the task-shifting of some entomologist responsibilities to community health workers (CHWs) or village health teams (VHTs). This study evaluated the unit cost of integrating VectorCam^TM^ into routine surveillance in Uganda.

**Methods:**

Data were extracted from a 12-month pragmatic parallel randomized trial conducted across two districts in Uganda from 2023 to 2024. This analysis accounted only for costs directly associated with field activities, including personnel, transport, and supplies. Three VectorCam^TM^ implementation models were analyzed. Implementation Model 1 represented full task-shifting of mosquito surveillance responsibilities to village health teams, where they were collected, analyzed, mosquito identified and reported data without the involvement of VCO and the use of VectorCam^TM^. Implementation Model 2 incorporated quarterly supervision by VCO to support village health team-led activities. Implementation Model 3 included monthly VCO supervision, aligning with routine surveillance workflows, to assess the cost implications of adding digital surveillance to existing protocols.

**Results:**

The annual cost of routine surveillance (control arm) per district was USD $10,615. In comparison, VectorCam^TM^ reduced costs to USD $8,852 in Model 1 (16.6% savings), USD $9,200 in Model 2 (13.3 savings), and USD $9896 in Model 3 (6.8% savings). These reductions were driven primarily by reductions in personnel and transport expenditures, although they were partially offset by higher supply costs associated with devices and service fees. The per-device analysis demonstrated that each VectorCam^TM^ unit represented an annual cost of USD 144.51 but replaced USD 438.29 worth of surveillance functions, resulting in net savings of USD 293.78 per device under Implementation Model 1. Sensitivity analyses demonstrated increased savings in scenarios with existing digital infrastructure or the individual usage of VectorCam^TM^ by one VHT.

**Conclusion:**

Digitalizing vector surveillance with VectorCam^TM^ offers a cost-saving and scalable approach for low- and middle-income countries. By shifting resources from specialized, labor-intensive methods such as microscopy to technology operable by CHW. VectorCam^TM^ supports more efficient data collection while reducing costs. These findings suggest that integrating digital task-shifting tools into national malaria surveillance programs can enhance vector surveillance in resource-constrained settings.

**Supplementary Information:**

The online version contains supplementary material available at https://doi.org/10.1186/s12936-026-06029-1.

## Background

### Importance of entomological surveillance

Among the 460 species in the *Anopheles* mosquito genus, only 30–40 serve as primary malaria vectors, with distributions varying regionally on the basis of environmental conditions [1]. This geographic specificity highlights the need for tailored malaria control strategies. Vector control, including long-lasting insecticide-treated bed nets (LLINs) and indoor residual spraying (IRS), plays a crucial role in combating mosquito-borne diseases and is among the highest economic returns in public health [2–5]. Surveillance is critical for adapting these control strategies to local contexts and identifying emerging threats, such as the spread of *Anopheles stephensi* from South Asia to East Africa. By tracking mosquito distribution, behavior, and feeding patterns, surveillance drives evidence-based interventions, optimal resource allocation, and intervention effectiveness monitoring [6].

### Current challenges in Uganda

In Uganda, malaria vector surveillance relies on mosquito collection across sentinel sites nationwide. Field teams deploy CDC light traps (Centers for Disease Control and Prevention model 1012), pyrethrum spray catches (PSCs), prokopak, and occasionally human landing catches (HLCs) to gather mosquito samples [7]. These samples are transported to a central district laboratory, where they are morphologically identified by entomologists or vector control officers (VCOs), and the data are subsequently digitized manually from paper-based records. VCOs have undergone at least three years of training in entomology and vector surveillance. Using light microscopy, they analyze specimens for species, sex, and abdominal status before sending a subset for molecular identification via polymerase chain reaction (PCR) or molecular sequencing [8]. Most national malaria elimination programs (NMEPs) continue to rely on this sentinel-site surveillance model despite two fundamental limitations: the national shortage of entomologists forces surveillance sites to be sparsely distributed yet treated as representative of entire regions, and the labor-intensive workflow creates substantial delays between specimen collection and data reporting [9, 10]. These delays hinder timely decision-making, undermining effective malaria control efforts. With faster data collection, health officials could promptly shift resources and change vector control strategies to address emerging vector threats.

### Development and validation of the VectorCam^TM^ technology

**Fig. 1:**
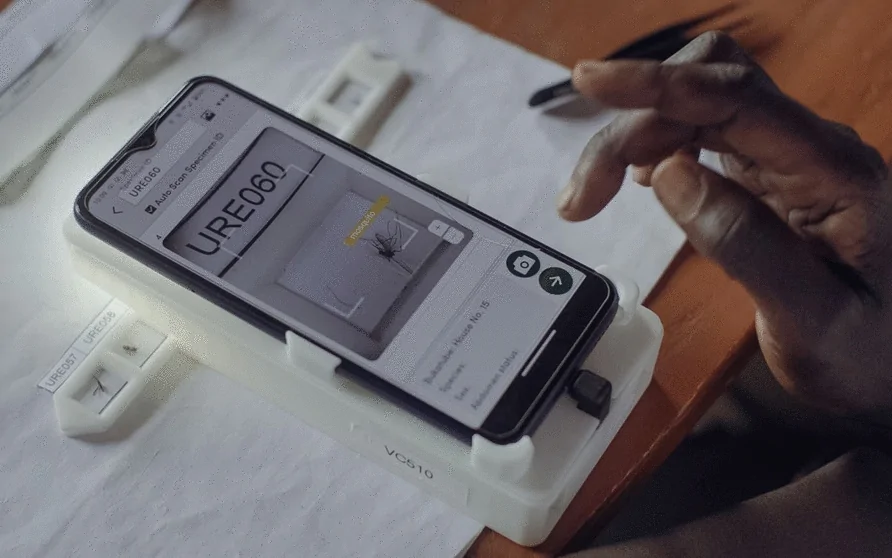
VectorCam, low-cost, portable, AI-powered tool for identifying malaria vectors

In recent years, the development of digital technologies and artificial intelligence have emerged as promising solutions to address the limitations of paper-based vector surveillance systems. To support these efforts, the Johns Hopkins University Center for Bioengineering Innovation and Design (CBID) developed VectorCam^TM^, an AI-enabled vector surveillance tool that uses computer vision algorithms to automate the morphological identification of mosquito vectors in field settings (Fig. 1). The VectorCam^TM^ platform supports rapid identification of mosquito species, sex, and abdominal status and pairs with a low-cost hardware attachment that connects to a smartphone for field use. The design of the tool was refined and validated through collaboration with more than 70 health technicians and vector control officers across Africa [9, 10]. VectorCam^TM^ uses convolutional neural networks that run locally on the smartphone, allowing the system to operate without internet connectivity and on a low-cost smartphone device. The algorithms are built to recognize the commonly encountered types of mosquito species monitored by surveillance programs, including *Anopheles funestus, An. gambiae, “An.* other” (secondary malaria vectors that may be further identifiable by microscopy and molecular techniques), as well as *Culex, Mansonia,* and *Aedes*. These algorithms identify an imaged mosquito’s species, sex, and abdomen status within seconds of taking a picture [9]. VectorCam^TM^ enables nonspecialist community members, such as village health teams (VHTs), to contribute to technical vector surveillance tasks, thereby expanding entomological surveillance capacity for national malaria elimination programs [10]. The system can be integrated with national databases to facilitate timely and responsive decision-making.

This study is part of a randomized trial conducted across two districts in Uganda between August 2023 and August 2024 to evaluate the efficacy and effectiveness of VectorCam^TM^ [11]. The trial was designed to assess whether VectorCam^TM^ could support accurate, timely, and complete entomological data collection when used by VHTs under routine programmatic conditions, as well as to evaluate the costs of incorporating VectorCam^TM^ into existing surveillance workflows. In this trial, VectorCam^TM^ achieved 93% classification accuracy across the major mosquito classification categories relevant to routine surveillance, including *Anopheles gambiae, Anopheles funestus, secondary Anopheles species (“Anopheles other”), Culex, Mansonia, Aedes,* and *non-mosquito* specimens. It reduced the median identification time to 17 s per mosquito, compared with 34.5 s using routine microscopy-based methods, and achieved near-complete data reporting, with approximately 100% data completion compared with 60% in routine surveillance. VHTs processed more than 77,000 mosquitoes during the study period, demonstrating the feasibility of community-based, AI-enabled surveillance. This trial also served as a pilot for a larger implementation study in the country, which began in February 2026 and is currently deploying the tool across fourteen districts [12].

This analysis examines the costs of integrating VectorCam^TM^ into Uganda’s malaria vector surveillance system relative to current, microscopy-based methods. We evaluate three VectorCam^TM^ implementation models with varying personnel and transportation structures, factors that directly influence overall cost, financial feasibility, and scalability.

Without rigorous cost data, digital innovations often remain confined to the pilot phases and are not integrated into routine health systems. By quantifying the financial implications of adopting VectorCam^TM^ under real-world field conditions, this analysis estimates the district-level cost of integration into Uganda’s malaria surveillance system. These findings provide essential evidence to inform decision-making regarding the incorporation of digital and AI-enabled tools into national malaria elimination program operations, particularly in low- and middle-income countries where surveillance investments must be balanced against other vector control priorities, such as long-lasting insecticide-treated nets and indoor residual spraying.

## Methods

### Study design and field procedures

Field cost data for this analysis were collected during a 12-month pragmatic parallel randomized trial conducted in two geographically distinct Ugandan districts: Mayuge (Eastern Region) and Adjumani (West Nile Region). In each district, three sentinel sites were selected on the basis of low, medium, and high malaria prevalence to capture environmental and epidemiological differences and provide diverse backgrounds for the VectorCam^TM^ field test. In total 6 sentinels surveillance sites participated in the trail and within each sentinel site, forty households were selected. District and household selection was conducted in collaboration with the Ugandan Ministry of Health and with technical experts from the National Malaria Elimination Division (NMED) and the Vector Control Division (VCD using routine surveillance criteria, including household willingness to participate, ecological diversity, and proximity to water bodies. All study protocols were approved by the Johns Hopkins University Institutional Review Board, Baltimore, MD, USA, and the AIDS Support Organization Institutional Review Committee, Uganda (IRB #00376201).

**Fig. 2:**
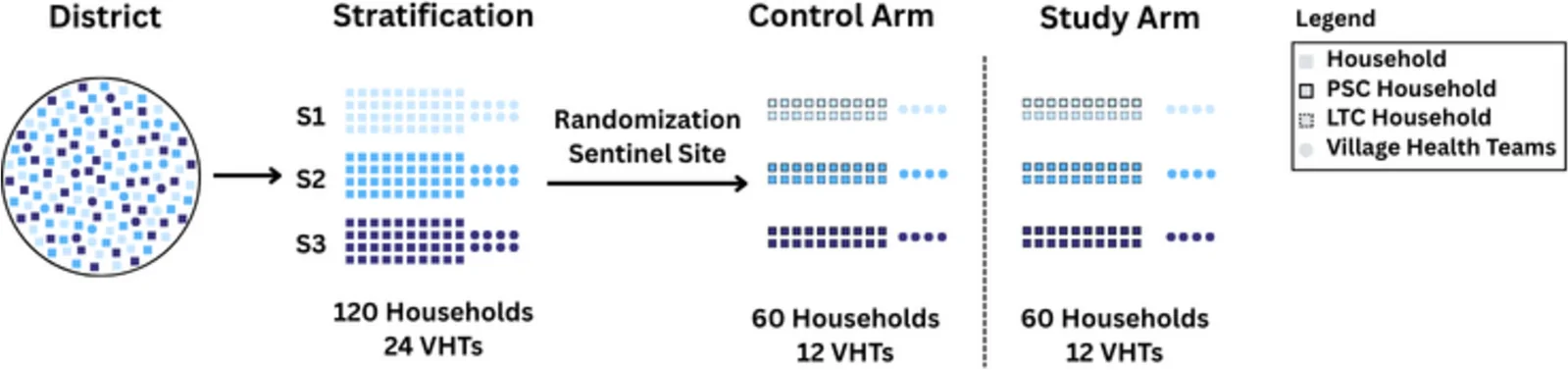
Study design and household randomization framework for the VectorCam^TM^ trial

**Fig. 3:**
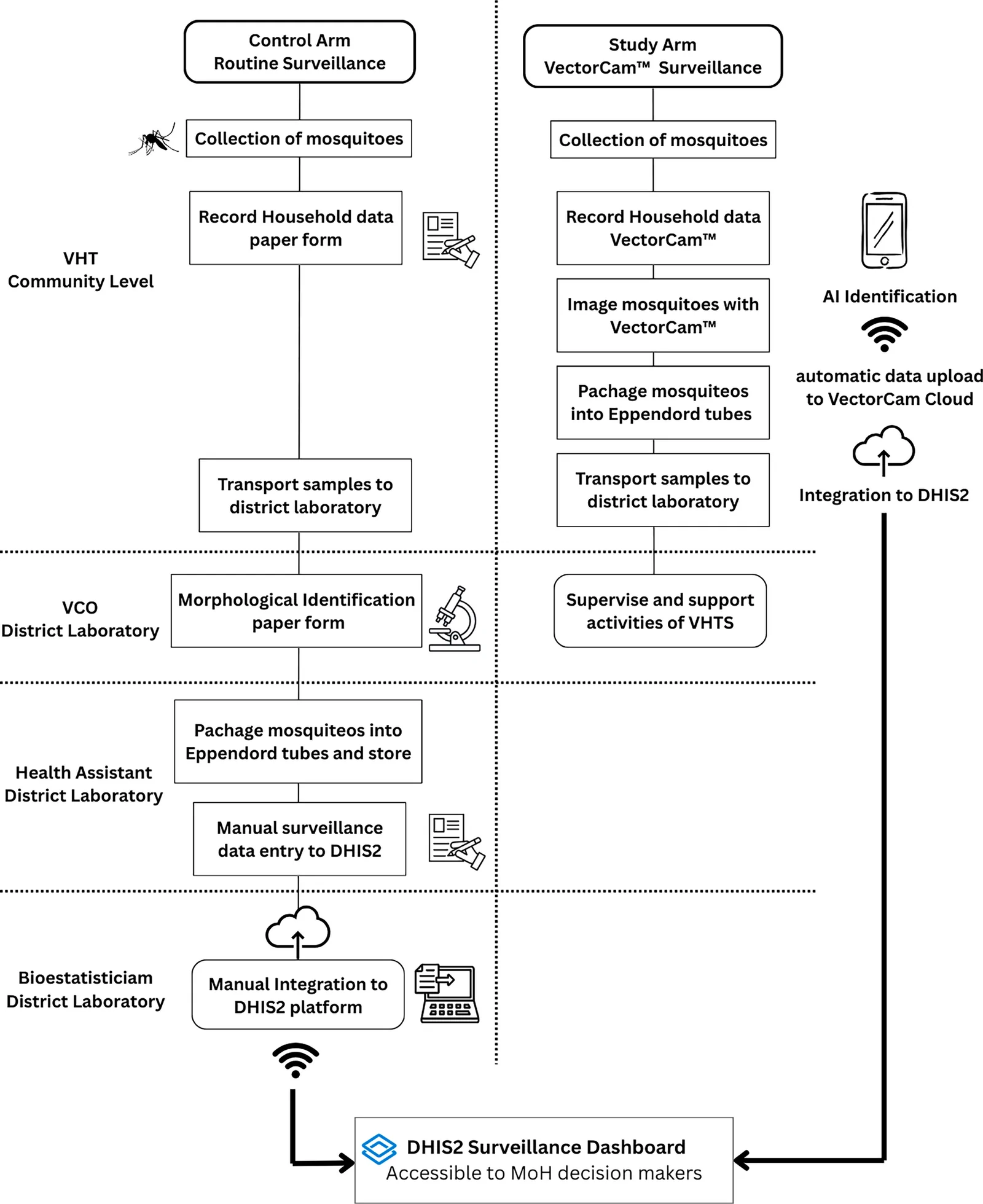
Surveillance workflows and personnel responsibilities in the control and study arms.

The study enrolled 240 households and 48 village health team members, who were randomized equally across the intervention and control arms (Fig. 2). All VHTs received foundational entomological surveillance training, with additional smartphone and application training provided to the intervention group. In both arms, mosquitoes were collected from 60 houses (30 using PSC, 30 with CDC light traps). In the control arm, VHTs packaged the mosquitoes and transported them to the district laboratory for morphological identification via VCOs. The data were recorded on paper and later entered the District Health Information Software 2 (DHIS2), Uganda’s national health data reporting system [13]. In the intervention group, VHTs used VectorCam^TM^ to identify species, sex, and abdomen status at the point of collection, with data from the app automatically integrated into DHIS2. For research purposes, all samples were also morphologically identified by VCOs and compared to the app results (Fig. 3).

### Collection and analysis of cost data

Actual financial costs were collected for personnel, transportation, and supplies directly involved in field activities at sentinel sites, from mosquito collection through data digitization onto DHIS2 (Fig. 3). Study-specific costs that would not occur during routine surveillance conditions were excluded, including research team travel to field sites, additional training conducted for research purposes, research supervision, and data analysis activities. Moreover, research, evaluation and economical-specific costs, such as morphological identification via PCR, were excluded. We used a “bottom-up” or “ingredient-based” costing approach from the health system perspective, drawing on both budgetary records and primary field data. Unit costs were obtained from project financial records, procurement data, and local market prices. All costs were collected in Ugandan shillings (UGX) and converted to U.S. dollars using an exchange rate of UGX 3,680 per USD (as of September 23, 2024) [14]. The data were input into a costing tool developed in Microsoft Excel 2021. Costs were analyzed at the district level, and capital costs were annualized, assuming equal annual allocation over the expected useful life of each piece of equipment.

To explore potential implementation pathways, we propose three implementation models for integrating VectorCam^TM^ into Uganda’s vector surveillance protocols. Implementation Model 1 assumes full task shifting of mosquito identification to VHTs without supervision from VCOs. Implementation Model 2 represents an intermediate approach in which VHTs conduct surveillance activities and identify mosquitoes with VectorCam with quarterly supervision from VCOs. Implementation Model 3 incorporates the same task-shifted model as implementation models 1 and 2 but includes monthly VCO supervision, which represents more supervision-intensive operations. Implementation Model 3 maintains monthly VCO supervision, mirroring the supervision intensity of current routine surveillance in Uganda (VCOs for five days each month). While our study arm followed the Implementation Model 3 structure because additional study-related responsibilities require VCOs, the implementation pathway is designed for districts to initially adopt Implementation Model 3 (monthly supervision by entomologists), then transition to Implementation Model 2 (quarterly supervision by entomologists), and ultimately progress to Implementation Model 1 (no supervision). This phased approach supports the gradual integration of VectorCam^TM^ with existing surveillance systems while allowing VHTs to develop the necessary skills for independent operation and transition to a full task-shifted mosquito identification implementation model. While the primary methodology involved a district-level cost analysis, an additional device-level analysis was conducted to assess the integration cost of individual VectorCam^TM^ units.

A sensitivity analysis was conducted to explore alternative scenarios relevant to programmatic scale-up. First, routine surveillance activities may, in some contexts, use exclusively CDC light traps rather than a combination of collection methods (e.g., PSC and CDC light traps), where the PSC represents a more resource-intensive component. Second, in some districts, smartphones and power banks may already be available through existing digital health platforms used by VHTs. Third, in certain implementation settings, VectorCam^TM^ may be deployed to individual users rather than pairs, with one VHT operating on a single device instead of two VHTs sharing one device. Accordingly, the sensitivity analysis examined (a) a routine surveillance model relying exclusively on CDC light traps; (b) the exclusion of smartphone and power bank costs; and (c) a comparison of paired (two VHTs per device) versus individual deployment (one VHT per device).

#### Personnel and transport

Personnel expenses were calculated per diem of UGX 12,000 (USD 5.4) for all staff, as specified in the Uganda NMCD surveillance budget. This analysis included only field staff directly involved in surveillance activities (Table 1). Payroll personnel, training staff, and overhead costs were excluded. Roles such as the director and deputy director of the NMCD, the data analyst for DHIS2, and the country’s lead entomologist were also omitted, as these roles were common to both arms.

**Table 1:** Field staff required routine surveillance and three VectorCam^TM^ implementation models

	Personnel per district (average days per month)			
	Routine surveillance	Implementation Model 1	Implementation Model 2	Implementation Model 3
VCO, responsibility	2 (5), Monthly Identification	0, No supervision	2 (1.67), Quarterly Supervision	2 (5), Monthly Supervision
VHT	12 (3)	12 (3)	12 (3)	12 (3)
Biostatistician	1 (8)			
Health assistant	3 (5)			

The VectorCam^TM^ system enables VHTs equipped with these devices to conduct monthly surveillance activities that also routinely require biostatisticians, VCOs, and health assistants. VectorCam^TM^’s automated identification replaces the need for microscope-based analysis by VCOs, reducing reliance on specialized expertise. In Implementation Models 2 and 3, VCOs shift to roles in oversight, quality assurance, and training for VHTs. In current routine surveillance, health assistants and village health teams collect paper-based field data that biostatisticians enter into the DHIS2 platform, whereas the VectorCam^TM^ system uploads surveillance data directly to the platform, eliminating these manual steps (Fig. 3).

Transportation covered commuting costs for VCOs and health assistants, following current surveillance practices. In accordance with the Ministry of Health guidelines, a standardized per diem UGX 20,000 (USD 5.26) was provided across districts regardless of distance traveled, mode of transportation, or fuel cost incurred. In the VectorCam^TM^ intervention, samples require only a single transport to the district laboratory for storage, which is conducted by either the VHT or VCO. This contrasts with current routine surveillance, which requires daily sample transport for ongoing morphological identification by VCOs.

#### Supplies

Supply expenses included all consumable and nonconsumable materials required to conduct entomological surveillance activities in both the control and study arms. These were categorized into (a) general surveillance supplies common in both arms. Within this category, a subcategory of collection methods was established to differentiate the operational costs between CDC light traps and pyrethroid spray catches (PSCs), the two primary trapping methods used in routine entomological surveillance in Uganda, and (b) arm-specific supplies. All costs are reported per district on a monthly and annual basis to allow comparisons across arms. VectorCam^TM^-related costs were estimated on the basis of study expenditures, with adjustments to reflect potential real-world implementation. Although the devices used in the study were assembled at the Johns Hopkins University Center for Bioengineering Innovation and Design (CBID), the analysis uses cost estimates based on local manufacturing and assembly, informed by data from local manufacturers. A VectorCam^TM^ service fee was incorporated to cover technical support and platform maintenance, as well as the cloud server and storage. Capital costs were annualized by distributing their value over their expected useful life: smartphones (3 years), power banks (5 years), CDC light traps (5 years), VectorCam devices (5 years) and laboratory microscopes (10 years).

## Results

### Unit cost analysis

In our 12-month randomized trial conducted across two districts in Uganda, we collected comprehensive data on personnel, transport, and supply expenditures from the control and study arms to compare the costs of routine surveillance with those of the three VectorCam^TM^ surveillance approaches (Table 2).

**Table 2:** Annual costs per district of surveillance activities, routine and VectorCam^TM^ surveillance implementation models

Annual Costs	Routine	Im*. Model 1	Im. Model 2	Im. Model 3
Personnel	$2700	$1409	$1539	$1800
Supplies	$6,676	$7,444	$7,444	$7,444
Transportation	$1239	$ -	$217	$652
Total	$10,615	$8,852	$ 9,200	$9,896

*Im Implementation

The annual cost of routine surveillance per district is estimated at USD 10,615. By comparison, the annual VectorCam^TM^ surveillance costs are estimated to be USD 8,852 (Implementation Model 1), USD 9,200 (Implementation Model 2), and USD 9,896 (Implementation Model 3).

Implementation Model 1, which implements complete task shifting to VHTs, results in a 16.6% reduction in total costs compared with current surveillance methods. With the addition of quarterly VCO supervision in Implementation Model 2, cost savings decreased to 13.3%. The Implementation Model 3 implementation, which maintains monthly VCO supervision protocols, produces marginal savings of 6.8%.

Annual district-level supply costs were estimated at USD 6,675.92 for routine entomological surveillance and USD 7,443.70 for the VectorCam^TM^ intervention, corresponding to an incremental cost of USD 767.78 per district per year. However, across both arms, most of the costs were driven primarily by pyrethrum aerosol spray (USD 2101.30, 32%), followed by monthly consumables, including masks, Eppendorf tubes and silica gel (2038.04, 31%). CDC light traps represented (USD 495.00, 8%); however, these costs did not quantify maintenance, changes in batteries or replacements. These expenses accounted for a large share of the total mosquito collection supplies common in both arms (USD 6,576.63 annually). These costs were identical across arms, as trapping methods were included within common surveillance supplies. VectorCam^TM^-specific supplies (USD 867.07) cost USD $767.78 more annually than do routine surveillance supplies ($99.29) across all three implementation models (Table 3).

**Table 3:** Supplies and associated costs per district for routine surveillance, VectorCam^TM^ intervention, and mosquito collection common to both

	Units/district	Recurrent Payment	Annual Cost (USD)
**Routine surveillance**			**$99.29**
Entomological surveillance questionnaire	12	Monthly	$19.57
Permanent markers fine tip	2	Quarterly	$32.61
Laboratory microscope	2	Every 10 years	$47.12
**VectorCam**^**TM**^ **intervention**			**$867.07**
Perforating sheets	1	Quarterly	$222.83
Phone	6	Every 3 years	$133.52
VectorCam^TM^ hardware	6	Every 5 years	$45.60
Power banks	6	Every 5 years	$7.37
Data phone plan (internet)	6	Monthly	$195.65
Cloud Server and Storage	1		$104.61
VectorCam^TM^ service fee	1	Monthly	$157.50
**Mosquito collection**			**$6576.63**
KN95 Masks (1), Eppendorf Tubes (2), Silica Gel (3)	*	Monthly	$2038.04
Petri Dish (6), Cotton Wool (3), Masking Tape (3), Small/Medium/Large Zip-Close Bags (3), Ethanol (6), Filter Papers (4), Markers (2)	*	Quarterly	$1045.54
Flashlights (6), Batteries (24), Ground Sheets (60), Forceps (30), Hand Lenses (6), Plastic Storage Container (6)	*	Bi-Annual	$896.74
CDC Light Traps	15	Every 5 years	$495.00
Pyrethrum aerosol spray	36	Monthly	$2101.30
**Total supplies routine surveillance**			**$6675.92**
**Total supplies VectorCam**			**$7443.70**
**Difference routine surveillance-VectorCam supplies**			**$767.78**

*Units are specified for each item. Some items may not be provided as single units and can be supplied in batches

The significance of the bold values are totals or subtotals

**Fig. 4:**
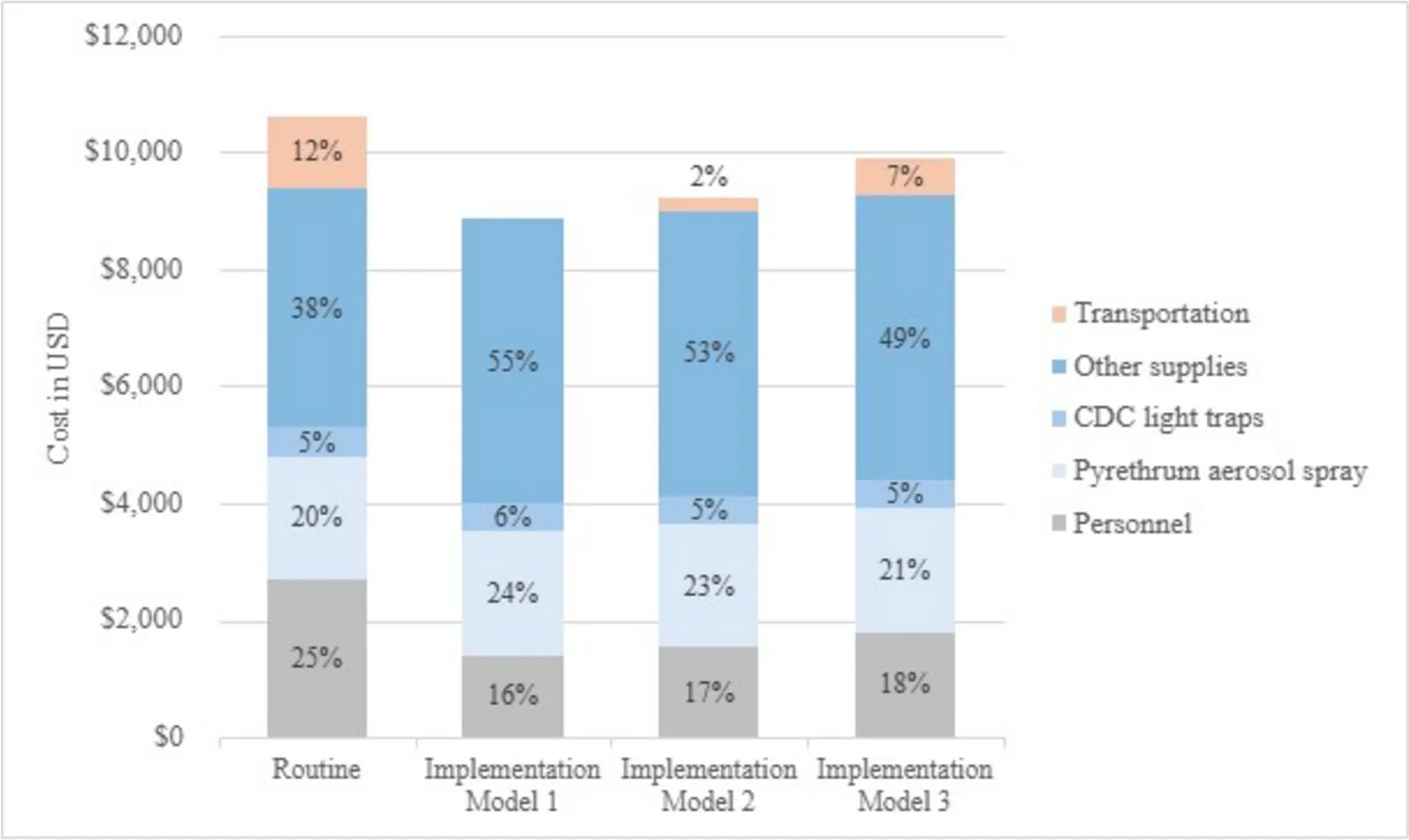
Total annual costs and proportional cost breakdown by surveillance implementation models. Distribution of personnel, supplies, and transportation costs for routine VectorCam^TM^ surveillance implementation models

Figure 4 illustrates how cost allocations shift across implementation models. Compared with routine surveillance, all VectorCam^TM^ implementation models allocate a substantially greater proportion of costs to supplies (75–84% versus 63%) and lower proportions to personnel (16–18% versus 25%). Transportation expenses are eliminated in Implementation Model 1 and reduced to just 2–7% in Implementation Models 2 and 3, compared with 12% in routine surveillance.

A sensitivity analysis assuming the use of CDC light traps only in both arms was conducted to assess the impact of removing pyrethrum spray catches (PSCs), given their high cost. Under this scenario, mosquito collection was adapted by replacing the 36 PSC cans per district and doubling the number of CDC light traps to maintain household coverage. This adjustment eliminated the need for PSC-related supplies, including KN95 masks and ground sheets. The primary cost driver under routine collection methods was the pyrethrum aerosol insecticide (36 cans totaling USD 2101.30), whereas the cost of CDC light traps increased from USD 495 to USD 990 when the number of traps doubled (Tables 4 and 5).

**Table 4:** Supplies associated with the combined collection implementation model versus only CDC light traps

Materials	Combine Im. Model, PSC and CDC light trap cost (USD)	CDC light traps Im. Model cost (USD)
KN95 Masks (1)*, Eppendorf Tubes (2), Silica Gel (3)	$ 2038.04	$ 1956.52
Petri Dish (6), Cotton Wool (3), Masking Tape (3), Small/Medium/Large Zip-Close Bags (3), Ethanol (6), Filter Papers (4), Markers (2)	$ 1045.54	$ 1045.54
Flashlights (6), Batteries (24), Ground Sheets (60)*, Forceps (30), Hand Lenses (6), Plastic Storage Container (6)	$ 896.74	$ 163.04
CDC Light Traps (15)	$ 495.00	$ 990.00
Pyrethrum aerosol spray (36)	$ 2101.30	$ –
**Mosquito collection supplies**	**$ 6576.63**	**$ 4155.11**
**Total supplies routine surveillance**	**$** **6675.92**	**$** **4254.40**
**Total supplies VectorCam**	**$** **7443.70**	**$** **5022.18**

The significance of the bold values are totals or subtotals

**Table 5:** Annual costs per district of surveillance activities adapted to different collection methods

Surveillance implementation models	Total cost, combined PSC and CDC light trap	Percent saving (%)	Total cost, CDC light trap only	Percent saving (%)
Routine surveillance	$ 10,615.05		$ 8194	
Implementation Model 1	$ 8,852.40	16.6	$ 6431	21.5
Implementation Model 2	$ 9,200.23	13.3	$ 6779	17.3
Implementation Model 3	$ 9,895.88	6.8	$ 7474	8.8

Under the CDC light trap-only scenario, eliminating PSCs substantially reduced mosquito collection costs from USD 6576.63 to USD 4155.11. This reduction was driven primarily by the removal of PSC-related consumables and insecticides. While the cost of CDC light traps doubled, this increase was minimal compared with the savings from eliminating PSC. At the district level, total surveillance costs decreased across all the implementation models, with cost savings increasing from 16.6 to 21.5% in Implementation Model 1, from 13.3 to 17.3% in Implementation Model 2, and from 6.8 to 8.8% in Implementation Model 3.

### VectorCam^TM^ per-device cost analysis

To evaluate VectorCam^TM^ implementation costs at the per-device level, we conducted a sensitivity analysis under alternative implementation scenarios. We analyzed the technology expenses and the surveillance costs it replaces. Each VectorCam^TM^ device represents an annual investment of USD 144.51, which includes hardware, accessories, service and cloud fees. Under the complete task-shifting model (Implementation Model 1), this technology replaces approximately USD 438.29 of annual surveillance functions, resulting in net savings of USD 293.78 per device per year in each surveillance district.

When smartphone and power bank costs were excluded, assuming that these resources were already available through existing digital health platforms in the district, the annual VectorCam^TM^ device cost decreased from USD 144.51 to USD 121.03 per device. Under this scenario, net annual savings increased from USD 293.78 to USD 317.26 per device. This reflects settings where a digital infrastructure has already been established, which is often the case when there are preexisting digital health interventions with mobile devices already provided, these preexisting resources can be leveraged for VectorCam^TM^ use, reducing the upfront costs of implementation [15].

The final scenario analyzed the implementation of VectorCam^TM^ under an individual device allocation, in which a single VHT operates VectorCam^TM^, rather than a pair of VHTs using a VectorCam^TM^. In this scenario, the annual device cost remained USD 144.51 per device, whereas the estimated annual surveillance cost offset increased from USD 438.29 to USD 555.68, resulting in net annual savings of USD 411.17 per device. This increase reflects the additional personnel-related efficiencies achieved when VectorCam^TM^ enables a single user to perform tasks that would otherwise require multiple personnel.

Finally, under a combined scenario where both assumptions were applied, where digital infrastructure at the district level was already established (i.e., phones and power banks available to use) and VectorCam is operated by a single VHT, the potential cost savings would be maximized, representing the most efficient and optimized implementation models for integrating VectorCam^TM^ into routine surveillance systems (Table 6).

**Table 6:** Sensitivity analysis of VectorCam^TM^ under digital infrastructure and one single-user scenario

Cost category	Item	Annual cost per device (USD)	No phone/no power bank	1 VHT/device
Cost offset by VectorCam	VCO (personnel)	$65.22	$65.22	$65.22
	VCO (transportation)	$108.70	$108.70	$108.70
	Biostatistician	$52.17	$52.17	$52.17
	Health assistant (personnel)	$97.83	$97.83	$97.83
	Health assistant (transport)	$97.83	$97.83	$97.83
	Surveillance Questionnaire	$3.26	$3.26	$3.26
	Laboratory microscope	$7.85	$7.85	$7.85
	Permanent markers	$5.43	$5.43	$5.43
	1 VHT (personnel)	–	–	$117.39
	**Total cost offset**	**$438.29**	$**438.29**	**$555.68**
VectorCam device costs	Phone	$22.25	–	$ 22.25
	VectorCam hardware	$7.60	$7.60	$7.60
	Power bank	$1.23	–	$1.23
	Data phone plan	$32.61	$32.61	$32.61
	VectorCam service fee	$26.25	$26.25	$26.25
	Cloud server and storage	$17.43	$17.43	$17.43
	Perforating sheets	$37.14	$37.14	$37.14
	**VectorCam device cost**	**$144.51**	**$121.03**	**$144.51**
Net annual savings per device		**$ 293.78**	**$317.26**	**$411.17**

The significance of the bold values are totals or subtotals

## Discussion

This study evaluated the cost of implementing a novel community-based approach to entomological vector surveillance via VectorCam^TM^ within routine malaria surveillance systems in two districts in Uganda. We assessed three implementation models that varied in the level of supervision by VCOs at the district level (Implementation Model 1: no VCO supervision; Implementation Model 2: quarterly supervision; Implementation Model 3: monthly supervision) and additionally conducted a sensitivity analysis to estimate cost offsets per device. This district-level cost analysis demonstrated that integrating VectorCam^TM^ into Uganda’s malaria vector surveillance system is associated with cost reductions across multiple implementation models compared with routine surveillance microscopy-based workflows. The cost savings ranged from 6.8% to 16.6% per district depending on the level of supervision, with greater reductions observed under fully task-shifted conditions (Implementation Model 1). The annual surveillance costs decreased by 16.6% per district, with smaller savings observed under supervisory implementation models (13.3% cost-saving Implementation Model 2 and 6.8% cost-saving Implementation Model 3). These reductions were driven primarily by decreased personnel time and transportation requirements, which were partially offset by technology-related supply costs.

A key finding of this study is that the cost structure of vector surveillance is largely driven by mosquito collection activities, which remained consistent across all the implementation models. For example, mosquito collection activities, including trap deployment, field consumption and specimen handling, remained the dominant cost component across all implementation models managed primarily by VHTS. In contrast, cost reductions were primarily observed in the “expertise” component of surveillance, including morphological identification, data entry, and supervisory functions. We observed that the VectorCam^TM^ device allowed for automated image-based species identification, reducing the time VCOs spent performing microscope-based morphological classification. Consequently, fewer sample transfers and reduced supervisory travel lowered transportation expenditures. Additionally, direct digital data capture and synchronization of surveillance data to DHIS2 eliminated the manual transcription of paper forms and reduced the need for biostatisticians to enter surveillance data into the Ministry of Health platform. By enabling image-based mosquito identification at the point of collection, VectorCam^TM^ reduces reliance on specialized personnel while preserving core field surveillance operations. Responsibilities routinely performed by trained entomologists are redistributed to VHTs supported by artificial intelligence.

Routine surveillance relies on centralized laboratory workflows, multiple types of expertise from different personnel, and repeated specimen transport. In contrast, VectorCam^TM^ facilitates decentralized, field-based identification and direct digital reporting, reducing workflow complexity and operational delays. This distinction highlights that digital tools do not eliminate surveillance activities but rather reorganize how these activities are performed within the health system. These findings are consistent with those of prior studies, emphasizing that task shifting has emerged as an important strategy for addressing health workforce shortages in low- and middle-income countries (LMICs). However, although VectorCam^TM^ enables task shifting of routine mosquito identification activities, it is not intended to replace entomological expertise. Rather, by automating routine classification tasks, this technology might allow trained entomologists to focus on higher-value activities such as quality assurance, molecular validation, surveillance planning, workforce training and supervision, and the investigation of emerging vector threats. Therefore, VectorCam^TM^ can complement existing expertise while expanding surveillance capacity. By redistributing tasks traditionally performed by trained professionals such as entomologists to CWHs or VHT, health systems have achieved improved coverage and reduced costs [16], demonstrating the economic benefits of task shifting in low- and middle-income countries. For example, a systematic review examining the cost implications of task shifting in LMICs revealed that 30 of 34 studies demonstrated that their interventions resulted in cost savings in their operations or health systems [17]. Specifically, a study in rural Zambia showed that engaging Community Health Workers in household mosquito trapping and genus-level sorting significantly reduced surveillance costs. The reported savings include an 83–96% reduction in cost per *Anopheles funestus* specimen captured [18]. Our findings extend this evidence by demonstrating that artificial intelligence can further enhance task shifting by enabling nonspecialists to perform technically complex identification tasks. In our analysis, a 16.6% reduction in annual district surveillance costs represents meaningful operational savings within constrained public health budgets. On the basis of an annual routine surveillance cost of USD 10,615.05 per district, this translates to approximately USD 1762.66 saved per district per year under full task-shifting conditions. If implemented across Uganda’s districts, such reductions could generate substantial cumulative savings while maintaining surveillance capacity.

Furthermore, sensitivity analyses demonstrated that the cost-saving potential of VectorCam^TM^ is robust across different implementation scenarios. When digital infrastructure in the district, such as existing smartphones and power banks, is assumed to be available, device-related costs decrease, and net savings increase [19]. Similarly, under an individual-use implementation model in which a single VHT operates on each device, cost savings increased significantly owing to additional reductions in personnel requirements. This sensitivity analysis suggests that the economic benefits of VectorCam^TM^ might be greater in settings where digital infrastructure is already established and the tool can be easily incorporated or where surveillance activities are conducted by individual frontline workers.

Importantly, mosquito collection remained the primary cost driver across all implementation models, with PSC (USD 2101.30) and CDC light traps (USD 495) contributing substantially and remaining constant across approaches. A sensitivity analysis assuming the use of CDC light traps only demonstrated that limiting mosquito collection to this method, rather than the combined approach currently used in Uganda, can significantly reduce overall surveillance costs across both the study and control arms. Under this scenario, cost savings increased across all VectorCam^TM^ implementation models, with savings increasing from 16.6 to 21.5% in Implementation Model 1, from 13.3 to 17.3% in Implementation Model 2, and from 6.8 to 8.8% in Implementation Model 3 at the district level. These findings suggest that further cost reductions in surveillance systems will depend on innovations in mosquito collection methods. For example, investing in low-cost trapping solutions designed using locally available resources, similar to the VectorCam^TM^ approach, could reduce collection costs and contribute to a more cost-efficient and sustainable surveillance infrastructure.

This study has several limitations. First, our cost estimates are based on Uganda’s surveillance structure and may not be generalizable to countries or settings with different labor costs, infrastructures, or surveillance protocols. Second, the analysis focused on direct implementation costs and did not capture downstream economic impacts, such as changes in vector control effectiveness or healthcare costs associated with delayed or suboptimal surveillance. In practice, the cost of improper intervention planning, or the lack of intervention effectiveness monitoring, is far greater than the actual cost savings in the district. Third, national-level scale-up costs, including VectorCam platform training, system integration, and long-term maintenance, were not fully modeled in the current analysis and should be considered in future implementation research. Although VectorCam training is expected to be incorporated into existing National Malaria Control Division training structures as an additional module during national rollouts, rather than implemented through a separate parallel training system, these activities will still require dedicated resources that were not captured in the current analysis. This integrated approach may reduce disruption to routine NMED training operations while ensuring that VHTs, district teams, and national-level staff receive the necessary platform-specific training. Finally, this study represents a cost analysis of two districts in Uganda, rather than a full cost-effectiveness evaluation and does not include health outcomes such as cases averted or disability-adjusted life years as a result of more effective interventions driven by robust entomological surveillance data.

Future research should evaluate the broader economic impact of integrating VectorCam^TM^ into surveillance systems, including cost-effectiveness analyses that incorporate epidemiological outcomes and budget impact assessments at the national scale. Moreover, multi-country studies are important for assessing generalizability across diverse health system contexts. In addition, implementation research is critical for evaluating long-term usability, supervision requirements, training and maintenance costs at large scale and the transition from pilot implementation to sustained national deployment.

In summary, integrating VectorCam^TM^ into routine malaria vector surveillance was associated with district-level cost savings under task-shifting conditions while preserving core surveillance activities. These findings suggest that task-shifted, digitally enabled surveillance implementation models represent a viable pathway for strengthening vector surveillance systems in resource-constrained settings. By augmenting the capabilities of frontline health workers with artificial intelligence, tools such as VectorCam^TM^ offer a scalable and financially feasible approach to modernizing entomological surveillance.

## Conclusions

This study demonstrated that integrating VectorCam^TM^ into routine malaria vector surveillance in two districts in Uganda can reduce district-level surveillance costs while maintaining core surveillance activities. Depending on the implementation model, annual cost savings ranged from 6.8 to 16.6%, with the greatest reductions observed under fully task-shifted conditions where VHTs conducted surveillance activities with minimal VCO supervision. Cost savings were driven primarily by reductions in personnel time, transportation requirements, and manual data management, although these were partially offset by technology-related supply costs.

In the global fight against malaria, improving vector surveillance is critical for effective vector control. These findings suggest that AI-enabled surveillance technologies can improve the financial sustainability of vector surveillance programs while preserving surveillance capacity. The integration of digital tools that facilitate the rapid identification of mosquitoes and the automated, centralized reporting of mosquito population diversity, density, and dynamics provides a financially feasible approach to advancing surveillance systems, particularly in settings where entomologist shortages limit coverage and manual processes create labor-intensive workflows that delay data reporting.

## Supplementary Information

Supplementary Material 1.

## Data Availability

All data supporting the findings of this study are included within the article and its Supplementary Information (Additional file 1). Further details regarding the cost analyses and models used are available from the corresponding author upon reasonable request.

## Abbreviations

LTC: Centers for Disease Control and Prevention Light Trap (CDC light traps)
NMEP: National Malaria Elimination Programs
NMEDs: National Malaria Elimination Division
PSC: Pyrethrum Spray Catches
VCOs: Vector Control Officers
VHTs: Village Health Teams
AI: Artificial Intelligence
